# miR‐193a/b‐3p relieves hepatic fibrosis and restrains proliferation and activation of hepatic stellate cells

**DOI:** 10.1111/jcmm.14210

**Published:** 2019-04-03

**Authors:** Baoling Ju, Ying Nie, Xufang Yang, Xiaohua Wang, Fujuan Li, Meng Wang, Chuang Wang, Hongjun Zhang

**Affiliations:** ^1^ Department of Immunology Mudanjiang Medical College Mudanjiang Heilongjiang People's Republic of China; ^2^ Department of Pathophysiology Mudanjiang Medical College Mudanjiang Heilongjiang People's Republic of China; ^3^ Department of Pathogen Biology Mudanjiang Medical College Mudanjiang Heilongjiang People's Republic of China

**Keywords:** HSC activation, liver fibrosis, miR‐13b‐3p, miR‐193a‐3p

## Abstract

MicroRNAs (miRNAs) have been confirmed to participate in liver fibrosis progression and activation of hepatic stellate cells (HSCs). In this study, the role of miR‐193a/b‐3p in concanavalin A (ConA)‐induced liver fibrosis in mice was evaluated. According to the results, the expression of miR‐193a/b‐3p was down‐regulated in liver tissues after exposure to ConA. Lentivirus‐mediated overexpression of miR‐193a/b‐3p reduced ConA‐induced liver injury as demonstrated by decreasing ALT and AST levels. Moreover, ConA‐induced liver fibrosis was restrained by the up‐regulation of miR‐193a/b‐3 through inhibiting collagen deposition, decreasing desmin and proliferating cell nuclear antigen (PCNA) expression and lessening the content of hydroxyproline, transforming growth factor‐β1 (TGF‐β1) and activin A in liver tissues. Furthermore, miR‐193a/b‐3p mimics suppressed the proliferation of human HSCs LX‐2 via inducing the apoptosis of LX‐2 cells and lowering the levels of cell cycle‐related proteins Cyclin D1, Cyclin E1, p‐Rb and CAPRIN1. Finally, TGF‐β1 and activin A‐mediated activation of LX‐2 cells was reversed by miR‐193a/b‐3p mimics via repressing COL1A1 and α‐SMA expression, and restraining the activation of TGF‐β/Smad2/3 signalling pathway. CAPRIN1 and TGF‐β2 were demonstrated to be the direct target genes of miR‐193a/b‐3p. We conclude that miR‐193a/b‐3p overexpression attenuates liver fibrosis through suppressing the proliferation and activation of HSCs. Our data suggest that miR‐193a‐3p and miR‐193b‐3p may be new therapeutic targets for liver fibrosis.

## 1 INTRODUCTION

Liver fibrosis caused by various chronic damage and extracellular matrix (ECM) deposition is occurred in the vast majority of chronic liver diseases,^1^, ^2^, ^3^ including chronic viral hepatitis, non‐alcoholic fatty hepatitis, alcoholic liver disease and primary biliary cirrhosis, etc. The activation of hepatic stellate cells (HSCs) is confirmed to take centre stage in ECM deposition.^4^, ^5^ Various cytokines, such as transforming growth factor‐β1 (TGF‐β1), platelet‐derived growth factor (PDGF) and tumour necrosis factor‐α, may stimulate the activation of HSC that manifests as proliferation and transdifferentiation of HSCs into fibrogenic myofibroblasts.^2^, ^6^, ^7^ Moreover, the activated HSCs further secrete cytokines, including TGF‐β, PDGF and HGF to maintain the activated state of themselves.^7^, ^8^ TGF‐β is also an effective pro‐fibrotic cytokine, which promotes the synthesis of ECM proteins.^9^ Blocking of TGF‐β signalling could partly alleviate liver fibrosis.^10^

MicroRNAs (miRNAs) are a kind of small non‐coding single‐strand RNAs that repress protein translation via pairing to the 3′ untranslated regions of their target mRNAs.^11^ Increasing evidence has demonstrated the pivotal roles of miRNAs in the pathogenesis of liver fibrosis.^12^, ^13^, ^14^ A research by Ma et  al reported that miR‐214 promoted the development of liver fibrosis via inducing the activation of HSCs.^15^ In addition, miR‐873‐5p has been demonstrated to be a marker for liver fibrosis and regulate the early stages of liver fibrosis.^16^ MiR‐351 has been confirmed to promote liver fibrosis via targeting the vitamin D receptor, and miR‐351 inhibition is recognized as a therapeutic intervention for fibrotic diseases.^12^ MiR‐193a and miR‐193b are members of miR‐193 family, which are well conserved in different species. MiR‐193a‐3p/miR‐193a‐5p and miR‐193b‐3p/miR‐193b‐5p are mature miRNAs produced by miR‐193a/miR‐193b. Roy et  al found that the expression level of miR‐193a‐3p was decreased not only in the liver tissues of patients with liver cirrhosis but also in CCL_4_‐induced liver fibrosis in mice.^17^ In addition, a previous study showed that the level of miR‐193b was down‐regulated in human liver fibrosis samples, as compared with normal liver samples.^18^ So miR‐193 family may participate in the regulation of liver fibrosis, whereas its potential mechanisms are unclear and worthy of further investigation.

Concanavalin A (ConA) is a common inducer of immune‐mediated liver injury. After intravenous injection of ConA, an immune‐related liver fibrosis could be detected in mice.^19^ In this study, the effect of miR‐193a/b‐3p on ConA‐induced liver fibrosis was evaluated in mice. Moreover, whether miR‐193a/b‐3p affected the viability and activation of HSCs was determined in human LX‐2 cells.

## 2 MATERIALS AND METHODS

### 2.1 Animals and induction of liver fibrosis in mice

Male BALB/c mice at 8‐10 weeks of age were obtained from Liaoning changsheng biotechnology co., Ltd. The mice were fed adaptively for 1 week. To induce liver fibrosis, the mice were injected with ConA (8 mg/kg) via tail vein once a week for six consecutive weeks. The mice were intraperitoneally injected with pentobarbital sodium (200 mg/kg) before, or 2, 4, 6 weeks after ConA administration (n = 4 for each time‐point), then the liver tissues were collected for further experiments. The animal protocol was approved by the Institutional Animal Care and Use Committee at the Mudanjiang Medical College, and complied with the Guidelines for the Care and Use of Laboratory Animals published by the US National Institutes of Health.

### 2.2 Overexpression of miR‐193a/b‐3p in mice

The mice were randomly divided into five groups (n = 8 per group): control, ConA, ConA + negative control lentivirus (LV‐NC), ConA + LV‐miR‐193a and ConA + LV‐miR‐193b. To overexpress miR‐193a/b‐3p, mice were injected twice with lentivirus containing miR‐193a/b (1 × 10^9^ TU/mL, 150 μL; Wanleibio, Shenyang, China) at 24 hours after the first and fourth ConA injections. The mice in control and LV‐NC groups were injected with saline and lentivirus containing NC sequences respectively. One week after the sixth ConA injection, the mice were killed by intraperitoneal injection of pentobarbital sodium (200 mg/kg). The serum samples and liver tissues were obtained for subsequent tests.

### 2.3 RNA isolation and quantitative real‐time PCR

Total RNAs were extracted using TRIpure reagent (BioTeke, Beijing, China) from liver tissues and LX‐2 cells. Then total RNAs were reversely transcribed into first‐strand cDNA using Super M‐MLV (BioTeke). Quantitative real‐time PCR was carried out using 2 × Power Taq PCR MasterMix (BioTeke) and SYBR Green (Solarbio, Beijing, China) on an Exicycler™ 96 real‐time quantitative thermal block (Bioneer, Daejeon, Korea). The primers used for PCR amplification were listed in Table 1. Expression values of mRNAs and miRNAs were normalized with β‐actin and U6 respectively. The relative expression levels were calculated by the 2^−ΔΔCT^ method.

**Table 1:** Oligonucleotide primer sets for real‐time PCR

Name	Sequence(5′‐3′)	Length
CAPRIN1 F	AGTTGAAACGGTTGAGGTG	19
CAPRIN1 R	GCATTTGTGCCATAAGGTC	19
COL1A1 F	ACGGCTCAGAGTCACCCA	18
COL1A1 R	CCTCCGGTTGATTTCTCATCATA	23
α‐SMA F	TCCCTTGAGAAGAGTTACGAGTT	23
α‐SMA R	ATGATGCTGTTGTAGGTGGTT	21
TGF‐β2 F	CATCCCGCCCACTTTCTAC	19
TGF‐β2 R	TCCGTTGTTCAGGCACTCT	19
β‐actin F	CTTAGTTGCGTTACACCCTTTCTTG	25
β‐actin R	CTGTCACCTTCACCGTTCCAGTTT	24
mmu‐miR‐193a‐3p F	GCGTGAACTGGCCTACAAAGT	21
mmu‐miR‐193a‐3p R	GTGCAGGGTCCGAGGTATTC	20
mmu‐miR‐193b‐3p F	GCTCTAACTGGCCCACAAAGT	21
mmu‐miR‐193b‐3p F	GTGCAGGGTCCGAGGTATTC	20
U6 F	CGCAAGGATGACACGCAAAT	20
U6 R	GTGCAGGGTCCGAGGTATTC	20

### 2.4 Assessment of serum hepatotoxicity indices

The serum levels of alanine aminotransferase (ALT) and aspartate aminotransferase (AST) were used to assess hepatotoxicity and detected by commercial detection kits (Sigma, Santa clara, CA), following the manufacturer's protocols.

### 2.5 Sirius red staining

The liver tissues were fixed with 10% formaldehyde, embedded in paraffin and cut into 5‐μm sections. Then the sections were stained in 1% Sirius red solution for 1 hour and observed under a microscope (Olympus, Tokyo, Japan) at a magnification of 100×.

### 2.6 Assessment of liver tissue indices

The contents of Actin A and TGF‐β1 in liver tissues were assessed by commercial ELISA kits (Boster, Wuhan, China) according to the manufacturer's instructions. The content of hydroxyproline in liver tissues was detected by hydroxyproline assay kit (Nanjing Jiancheng Bioengineering Institute, Nanjing, China).

### 2.7 Double immunofluorescence staining of desmin and PCNA

Liver tissues were embedded in paraffin and 5‐µm‐thick sections were prepared. The sections were placed on slides and heated at 60°C for 2 hours. Following dewaxing, rehydration, the slides were incubated in boiled antigen retrieval buffer for 10 minutes. Then slides were blocked with goat serum for 15 minutes at room temperature. For double labelling, slides were incubated with desmin antibody (1:50; Proteintech, Rosemont, IL) plus proliferating cell nuclear antigen (PCNA) antibody (1:50; Santacruz Biotechnology, Santa Cruz, CA) at 4°C overnight, followed by incubation with fluorescein isothiocyanate (FITC)‐conjugated anti‐mouse IgG (1:200; Beyotime, Haimen, China) and Cy3‐conjugated anti‐rabbit IgG (1:200; Beyotime) for 90 minutes at room temperature. Finally, the slides were counterstained with 4′,6‐diamidino‐2‐phenylindole (DAPI) and observed under a fluorescence microscope (BX53; Olympus).

### 2.8 Cell culture and treatments

LX‐2 cells were purchased from Shanghai Zhong Qiao Xin Zhou Biotechnology Co., Ltd, and maintained in dulbecco's modified eagle medium (Gibco, Grand Island, NY, USA) containing 10% foetal bovine serum (Hyclone, Logan, UT) at 37 °C, under a 5.0% CO_2_ atmosphere.

To regulate miR‐193a/b‐3p expression, LX‐2 cells were transfected with miR‐193a‐3p mimics/inhibitor, miR‐193b‐3p mimics/inhibitor (Genepharma, Shanghai, China) using Lipofectamine 2000 Reagent (Invitrogen, Carlsbad, CA) according to the instructions. To induce the activation of human HSCs, LX‐2 cells were treated with 5 ng/mL TGF‐β1 (Sino Biological Inc, Beijing, China), 25 ng/mL activin A (PeproTech, Rocky Hill, NJ) or combination of 2.5 ng/mL TGF‐β1 and 12.5 ng/mL activin A for 48 hours.

### 2.9 Terminal deoxynucleotidyl transferase‐mediated nick end labelling

The apoptosis of LX‐2 cells was detected using one step terminal deoxynucleotidyl transferase‐mediated nick end labelling (TUNEL) Apoptosis Assay Kit (Beyotime). Briefly, LX‐2 cells were placed on slides and permeabilized in 0.1% Triton X‐100 (Beyotime) for 5 minutes at room temperature. Then the slides were incubated with TUNEL reaction mixture at 37 °C for 60 minutes. The nuclei were stained with DAPI for 5 minutes. The slides were observed under a fluorescence microscope and images were taken.

### 2.10 Western blot

Total protein was isolated from LX‐2 cells using RIPA (Solarbio) and quantified using a BCA Protein Assay Kit (Solarbio). Then equal amounts of protein samples were separated on SDS‐PAGE and transferred to polyvinylidene difluoride membranes. The membranes were blocked with 5% skimmed milk for 60 minutes, and incubated with primary antibodies against CAPRIN1 (1:500; Proteintech), Cyclin D1 (1:1000; Bioss, Beijing, China), Cyclin E1 (1:1000; Proteintech), p‐RbS807 (1:1000; Bioss), TGF‐β2 (1:1000; Bioss), COL1A1 (1:1000; Bioss), α‐SMA (1:1000; Bioss), p‐Smad2^ser467^ (1:1000; Cell Signaling Technology, Trask Lane Danvers, MA), Smad2 (1:1000; Cell Signaling Technology), p‐Smad3^S423/S425^ (1:1000; Cell Signaling Technology), Smad3(1:1000; Cell Signaling Technology) and β‐actin (1:1000; Santacruz Biotechnology) at 4°C overnight. Goat anti‐rabbit IgG or goat anti‐mouse secondary antibodies (1:3000; Solarbio) were employed at 37°C for 60 minutes. The bands were visualized using electro‐chemi‐luminescence (ECL) Western Blotting Substrate (Solarbio). The protein expression was standardized to β‐actin and quantified using Gel‐Pro‐Analyzer software (Media Cybernetics, Rockville, MD).

### 2.11 Luciferase reporter assay

The 3′untranslated region (3′‐UTR) sequences containing the predicted wild‐type (WT) target gene and a mutant (MUT) variant were amplified by PCR and inserted into the pmirGLO Dual‐Luciferase miRNA target expression vector. 293T cells were seeded into 12‐well plates and cotransfected with 500 ng of plasmids, 25 pmol of miR‐193a‐3p mimics or miR‐193b‐3p or mimics NC using Lipofectamine 2000. After transfection for 48 hours, the cells were collected and luciferase activities were detected by Dual‐Luciferase® Reporter Assay System (Promega, Madison, Wisconsin) according to the manufacturer's instructions.

### 2.12 Statistical analysis

All experimental data are expressed as mean ± SD. One‐way ANOVA was performed to analyse data among multiple groups using GraphPad Prism 5 (GraphPad Software Inc, La Jolla, CA). A *P* value of less than 0.05 was considered statistically significant.

## 3 RESULTS

### 3.1 Expression of miR‐193a/b‐3p in liver tissues of mice after exposure to ConA

**Figure 1:**
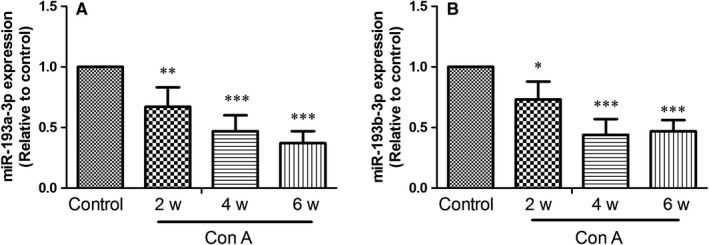
Expression of miR‐193a/b‐3p in liver tissues of mice after exposure to Concanavalin A (ConA). The expressions of miR‐193a‐3p (A) and miR‐193b‐3p (B) in liver tissues were detected by RT‐PCR at the indicated time‐points after treatment with ConA. All data were expressed as mean ± SD (n = 4). *P < 0.05, **P < 0.01, ***P < 0.001 vs the control group

Firstly, the altered expression of miR‐193a/b‐3p in liver tissues of ConA‐treated mice was assessed by RT‐PCR. As shown in Figure 1, there was a gradual decline in the expressions of miR‐193a‐3p (A) and miR‐193b‐3p (B) in liver tissues with the action time of ConA extending. So miR‐193a/b‐3p might participate in ConA‐induced liver fibrosis in mice.

### 3.2 Overexpression of miR‐193a/b‐3p attenuated ConA‐induced liver injury

**Figure 2:**
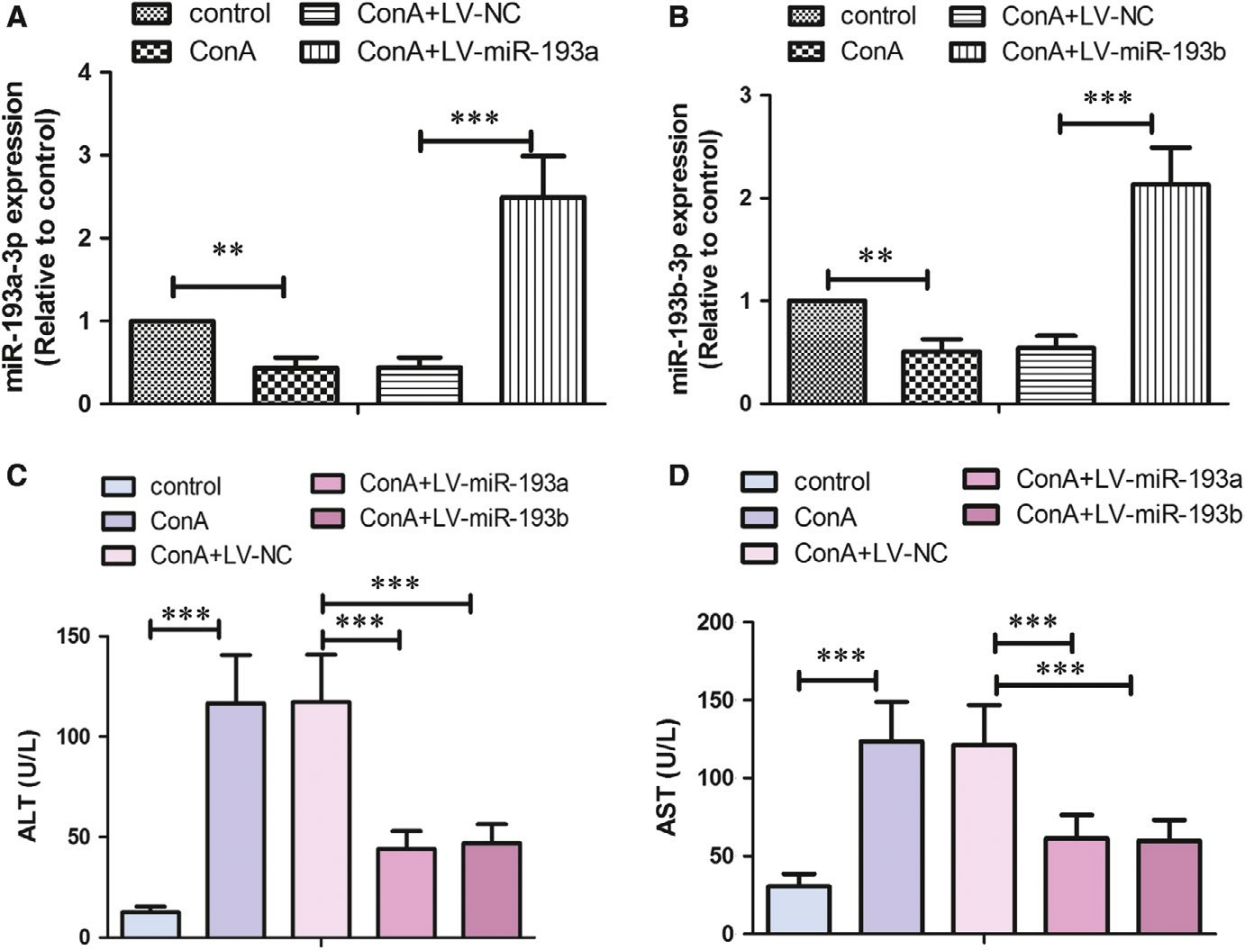
Effect of miR‐193a/b‐3p overexpression on Concanavalin A (ConA)‐induced liver injury. Lentivirus‐mediated overexpression of miR‐193a‐3p (A) and miR‐193b‐3p (B) in liver tissues was verified by RT‐PCR assay. The serum levels of alanine aminotransferase (ALT) (C) and aspartate aminotransferase (AST) (D) were assessed by commercial kits. All data were expressed as mean ± SD (for A & B, n = 6; for C & D, n = 8). **P < 0.01, ***P < 0.001 vs the indicated group

To enhance the expression of miR‐193a/b‐3p in liver tissues, the mice were injected with lentiviral particles containing miR‐193a/b‐3p. As presented in Figure 2A and B, ConA‐induced decreased expression of miR‐193a/b‐3p in liver tissues was significantly up‐regulated after infection of lentivirus. In kidney and heart tissues, the expression of miR‐193a/b‐3p was declined slightly after exposure to ConA, but the difference was not statistically significant (Figure S1). Lentivirus‐mediated overexpression of miR‐193a/b‐3p in kidney and heart tissues was also confirmed. Moreover, the serum levels of ALT and AST were increased by about 9.2 and 4.0 folds, respectively, after exposure to ConA for 6 weeks. Whereas, overexpression of miR‐193a/b‐3p could effectively reduce the ALT and AST levels (Figure 2C & D), suggesting that ConA‐induced liver injury was alleviated by enhancing miR‐193a/b‐3p expression.

### 3.3 Overexpression of miR‐193a/b‐3p relieved ConA‐induced liver fibrosis

**Figure 3:**
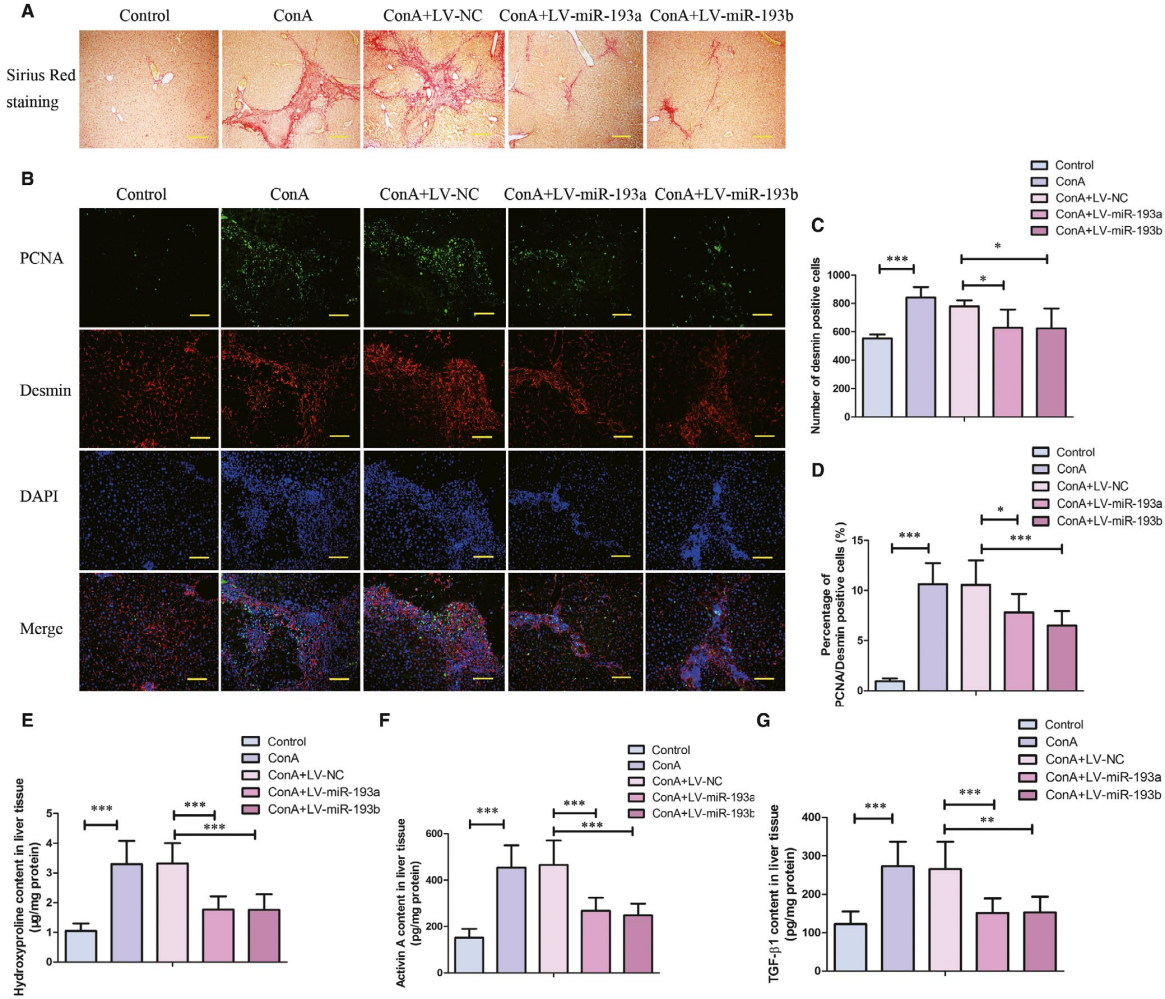
Effect of miR‐193a/b‐3p overexpression on Concanavalin A (ConA)‐induced liver fibrosis. A, Collagen formation in liver tissues was evaluated by Sirius red staining (100×). Scale bar is 200 μm. B, Expressions of desmin and proliferating cell nuclear antigen (PCNA) in liver tissues were determined by immunofluorescence staining (200×). Scale bar is 100 μm. Quantitative analyses of number of desmin positive cells (C) and percentage of PCNA/desmin positive cells (D) were shown. The contents of Hydroxyproline, activin A, and TGF β1 in liver tissues were detected by commercial kits. All data were expressed as mean ± SD (n = 8). *P < 0.05, **P < 0.01, ***P < 0.001 vs the indicated group

Marked fibrosis was observed by Sirius red staining in liver tissues after treatment with ConA for 6 weeks, which could be obviously restrained by lentivirus‐mediated overexpression of miR‐193a/b‐3p (Figure 3A). In addition, the expressions of desmin, a marker for HSCs, and PCNA were determined by double immunofluorescence staining. As shown in Figure 3(B‐D), the number of desmin positive cells was increased after exposure to ConA. However, overexpression of miR‐193a/b‐3p strikingly repressed desmin expression in liver tissues. The percentage of PCNA/desmin dual‐positive cells was increased by 10‐folds in ConA treatment group, which was decreased by overexpression of miR‐193a/b‐3p. Moreover, exposure to ConA resulted in increases in the contents of fibrosis‐related molecules hydroxyproline, activin A and TGF‐β1 in liver tissues, whereas miR‐193a/b‐3p overexpression restrained ConA‐induced increased levels of these molecules (Figure 3E‐G).

### 3.4 Effect of miR‐193a/b‐3p overexpression on apoptosis and cell cycle of human HSCs LX‐2

**Figure 4:**
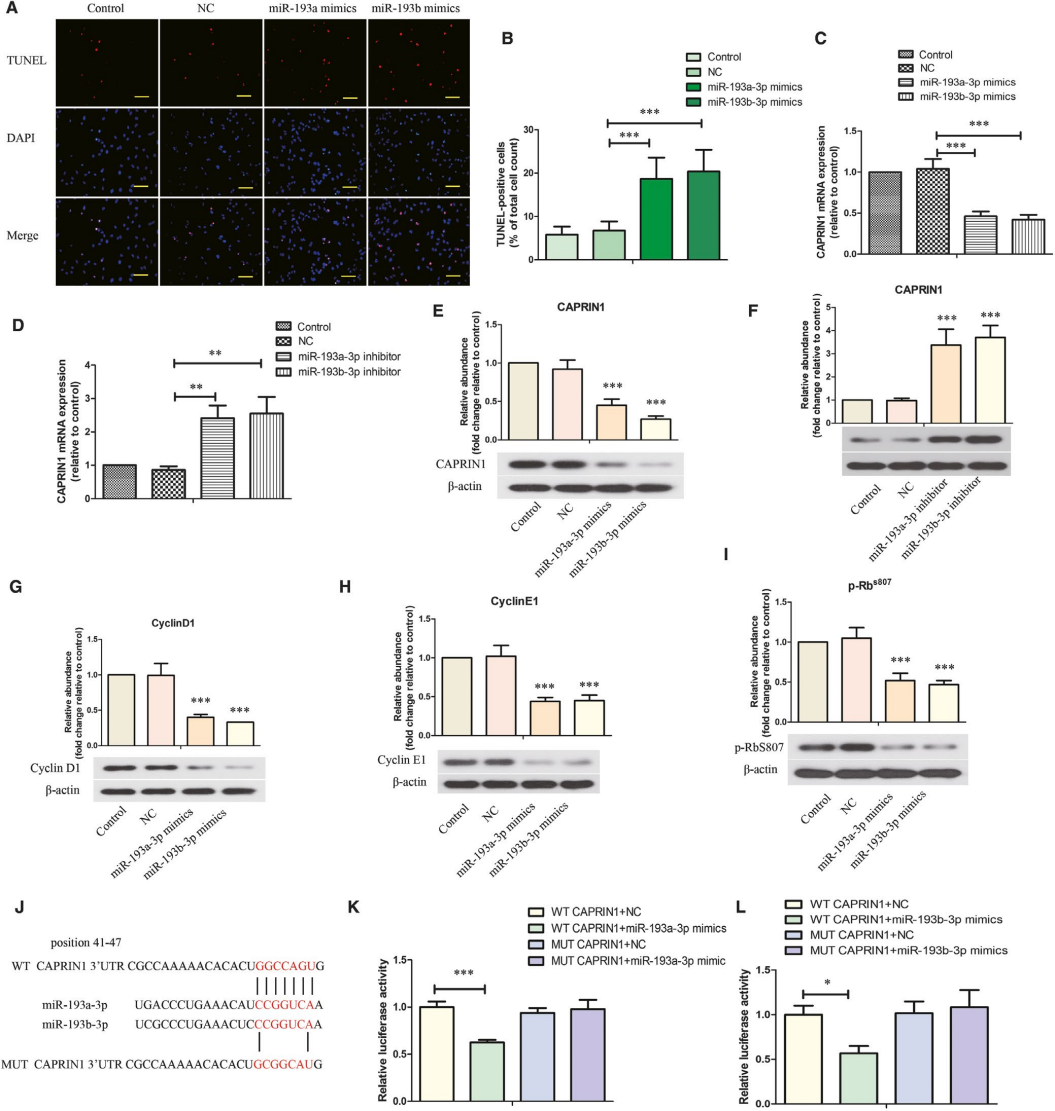
Effect of miR‐193a/b‐3p on apoptosis and cell cycle of human hepatic stellate cells LX‐2. (A,B) Apoptosis of LX‐2 cells were detected by Terminal‐deoxynucleotidyl transferase‐mediated nick end labelling (TUNEL) (200×) and the percentage of TUNEL‐positive cells was shown. Scale bar is 100 μm. (C,D) The mRNA expression of CAPRIN1 in LX‐2 cells was evaluated by RT‐PCR. The protein levels of CAPRIN1 (E,F), Cyclin D1 (G), Cyclin E1 (H) and p‐RbS807 (I) in LX‐2 cells were assessed by western blot analysis. J, The wild‐type (WT) and mutant (MUT) binding sites of the CAPRIN1 3′‐UTR to miR‐193a/b‐3p were cloned into the pmirGLO vector. The direct binding of miR‐193a‐3p (K) or miR‐193b‐3p (L) with CAPRIN1 was determined by dual‐luciferase report system. All data were expressed as mean ± SD (n = 3). *P < 0.05, ***P < 0.001 vs the indicated group

As illustrated in Figure 4A and B, overexpression of miR‐193a/b‐3p significantly induced apoptosis of LX‐2 cells as demonstrated by raising the percentage of TUNEL‐positive LX‐2 cells. The mRNA and protein expressions of CAPRIN1 were evidently inhibited by transfection of miR‐193a/b‐3p mimics, while promoted by miR‐193a/b‐3p inhibitor (Figure 4C‐F). Moreover, the protein levels of cell cycle‐related proteins Cyclin D1, Cyclin E1 and p‐Rb^s807^ were reduced by miR‐193a/b‐3p overexpression in LX‐2 cells. As shown in Figure 4G‐I, the ability of miR‐193a‐3p or miR‐193b‐3p in binding with CAPRIN1 was evaluated by dual‐luciferase report system. The results showed that in WT CAPRIN1+miR‐193a‐3p or miR‐193b‐3p group, the relative luciferase activity was significantly decreased. There was no difference between MUT CAPRIN1+NC and MUT CAPRIN1+miR‐193a‐3p or miR‐193b‐3p groups (Figure 4J‐L).

### 3.5 Overexpression of miR‐193a/b‐3p repressed the activation of human HSCs LX‐2 via targeting TGF‐β2

**Figure 5:**
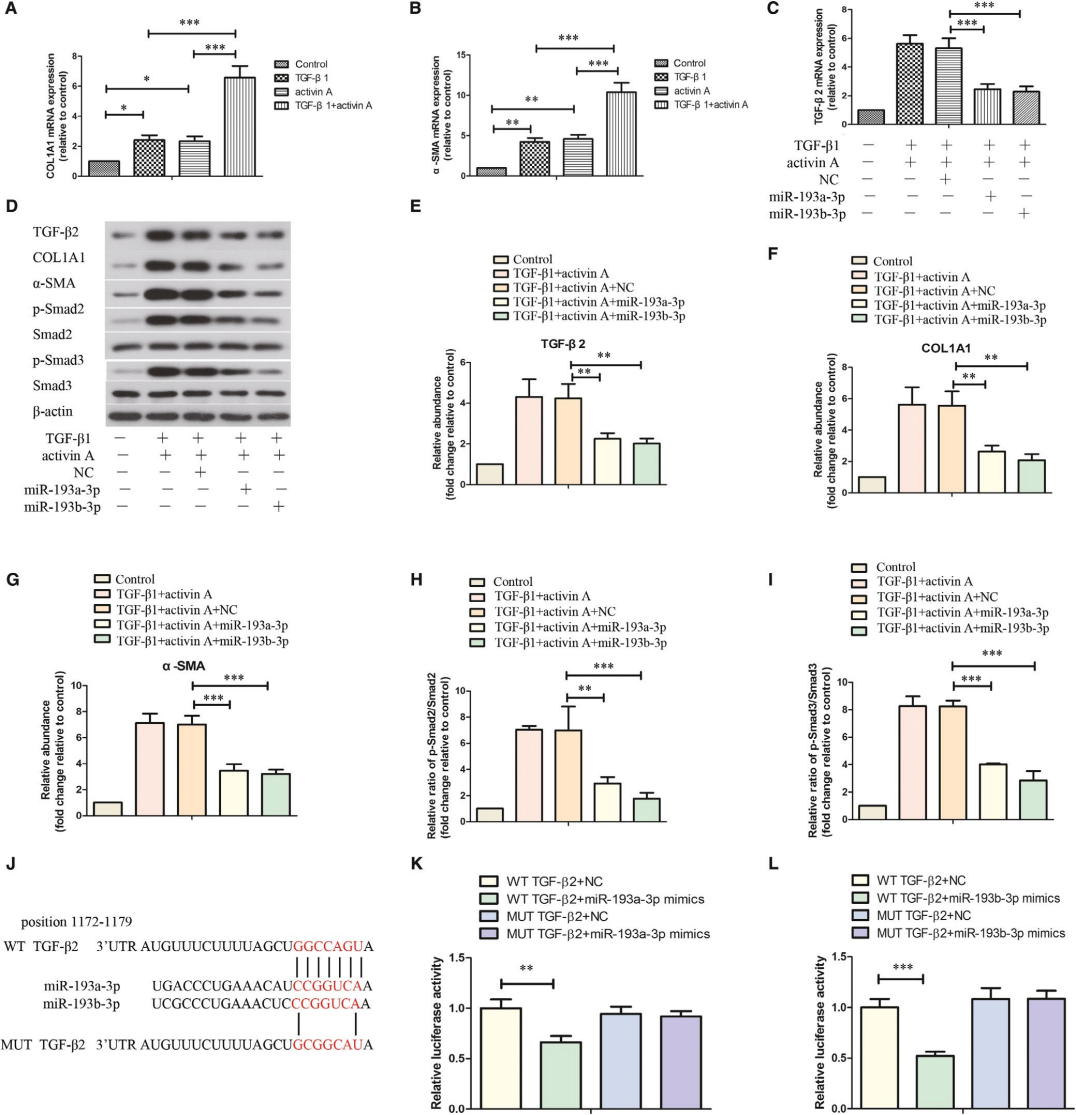
Effect of miR‐193a/b‐3p overexpression on activation of human hepatic stellate cells LX‐2. The mRNA expressions of COL1A1 (A) and α‐SMA (B) in LX‐2 cells that were treated with TGF‐β1, activin A or combination were assessed by RT‐PCR. C, The mRNA expression of TGF‐β2 in LX‐2 cells with different treatments was detected by RT‐PCR. D, The protein levels of TGF‐β2, COL1A1, α‐SMA, p‐Smad2, Smad2, p‐Smad3 and Smad3 in LX‐2 cells were evaluated by western blot assay. (E‐I) Quantitative analyses of the protein bands were shown. J, The wild‐type (WT) and mutant (MUT) binding sites of the TGF‐β2 3′‐UTR to miR‐193a/b‐3p were cloned into the pmirGLO vector. The direct binding of miR‐193a‐3p (K) or miR‐193b‐3p (L) with TGF‐β2 was determined by dual‐luciferase report system. All data were expressed as mean ± SD (n = 3). **P < 0.01, ***P < 0.001 vs the indicated group

To induce the activation of HSCs, LX‐2 cells were treated with TGF‐β1, or activin A, or combination of TGF‐β1 and activin A. As presented in Figure 5A and B, the mRNA expressions of COL1A1 and α‐SMA in LX‐2 cells were raised after treatment with TGF‐β1 or activin A, which were further promoted by combination of TGF‐β1 and activin A. So, we chose to induce the activation of LX‐2 cells by combination treatment with TGF‐β1 and activin A. As shown in Figure 5C, the mRNA expression of TGF‐β2 was promoted by co‐treatment with TGF‐β1 and activin A in LX‐2 cells, which could be inhibited by miR‐193a/b‐3p mimics. Moreover, TGF‐β1 and activin A‐induced increase in protein levels of TGF‐β2, COL1A1, α‐SMA, p‐Smad2 and p‐Smad3 in LX‐2 cells was restrained by overexpression of miR‐193a/b‐3p (Figure 5D‐I). As shown in Figure 5J‐L, the relative luciferase activity of LX‐2 cells was decreased by transfection with miR‐193a‐3p or miR‐193b‐3p in the WT TGF‐β2 groups, but not in the MUT TGF‐β2 groups. These results indicated that TGF‐β2 is the target gene of miR‐193a/b‐3p.

## 4 DISCUSSION

Repeated exposure to ConA has been demonstrated to resemble liver fibrosis during the pathological process of viral and autoimmune hepatitis.^20^ In this study, the mice received repeated 6‐week intravenous injection of ConA. Declined expression of miR‐193a/b‐3p was found in liver tissues after exposure to ConA. Lentivirus‐mediated overexpression of miR‐193a/b‐3p relieved ConA‐induced liver function injury and fibrosis in vivo. In addition, overexpression of miR‐193a/b‐3p induced apoptosis, restrained proliferation and activation of human HSCs LX‐2 cells in vitro. Our study for the first time suggested that miR‐193a/b‐3p overexpression protected against ConA‐induced liver fibrosis in mice.

The down‐regulation of miR‐193a‐3p and miR‐193b‐3p has been reported in human liver fibrosis samples.^17^, ^18^ The present study also demonstrated that miR‐193a/b‐3p expression was decreased after treatment with ConA. Why miR‐193a‐3p and miR‐193b‐3p are down‐regulated? One possible reason may be the phosphorylation of exportin‐5 (XPO5) that suppresses pre‐miRNA export from the nucleus.^21^, ^22^ However, the detailed mechanisms need to be further evaluated. Even so, we speculate that miR‐193a/b‐3p might play a major role in the regulation of liver fibrosis. To test this hypothesis, we enhanced the expression of miR‐193a/b‐3p using lentivirus in mice. The results showed overexpression of miR‐193a/b‐3p alleviated ConA‐induced liver injury as confirmed by reducing ALT and AST levels. In addition, Sirius red staining indicated that ConA‐induced liver fibrosis also relieved by miR‐193a/b‐3p overexpression.

Next, the exact mechanisms of miR‐193a/b‐3p to counteract liver fibrosis were investigated. Hepatic stellate cells are a kind of non‐parenchymal cells in liver, which are dedicated to hepatic development and homeostasis under the physiological conditions.^23^ But when liver injury occurs, activation of HSCs is a common consequence and contributes to liver fibrosis.^24^ To mitigate liver fibrosis, loss of activated HSCs is an effective measure.^25^ Desmin is one of the classical markers of HSCs.^26^ Our results showed that overexpression of miR‐193a/b‐3p suppressed ConA‐induced proliferation of HSCs as demonstrated by decreased expression of HSCs marker desmin and PCNA. It has been demonstrated that activated HSCs may result in accumulation of ECM and culminating in the formation of fibrosis tissue.^1^ Hydroxyproline is an important indicator of synthesis of collagen that is a key component of ECM. According to our results, ConA‐induced increase in hydroxyproline content was significantly reversed by miR‐193a/b‐3p overexpression. During the process of liver fibrosis, a lot of molecules are involved in the regulatory mechanisms. Activins and TGF‐βs belong to the TGF‐β superfamily and their abnormal high expressions have been found in fibrosis in multiple organs.^27^, ^28^, ^29^, ^30^ Previous study suggested that there was a potential interaction between TGF‐β and activin A, facilitating collagen production.^31^ Our previous research also indicated that TGF‐β1 and activin A levels were raised in ConA‐treated mice. In the present study, overexpression of miR‐193a/b‐3p relieved ConA‐induced liver fibrosis through decreasing TGF‐β1 and activin A levels.

Liver fibrosis is a reversible process and apoptosis, senescence and reversion from activated to quiescent stage of HSCs may provide possibilities. Inducing apoptosis of HSCs is an effective way to reduce the number of activated HSCs during liver fibrosis reversal.^32^ The apoptosis of HSCs also contributes to the degradation of ECM.^33^ Our results indicated that overexpression of miR‐193a/b‐3p significantly induced apoptosis of HSCs. CAPRIN1 suppression has been confirmed to inhibit cell proliferation via blocking cell cycle progression.^34^ CAPRIN1 is predicted to be a target gene of hsa‐miR‐193a/b‐3p and mmu‐miR‐193a/b‐3p. Cyclin D1 is also predicted to be a target gene of hsa‐miR‐193a/b‐3p, but was not for mmu‐miR‐193a/b‐3p (one binding site is mutated). So CAPRIN1 was focused in this study. Our results showed that miR‐193a/b‐3p could inhibit the mRNA and protein expression of CAPRIN1 via binding to its 3′‐UTR. Since Cyclin D1, Cyclin E1 and the Retinoblastoma protein Rb are the regulatory proteins that control G1 to S‐phase transition during the cell cycle, we also detected the effect of miR‐193a/b‐3p on the expression of these proteins. According to the results, overexpression of miR‐193a/b‐3p also decreased the protein levels of Cyclin D1, Cyclin E1 and p‐Rb, which suggested that the cell cycle of LX‐2 was blocked. However, whether miR‐193a/b‐3p regulates the expression of these proteins via direct binding to their 3′‐UTR or other mechanisms needs to be investigated in the future. Nevertheless, these results provide evidence that overexpression of miR‐193a/b‐3p inhibits the proliferation of human HSCs via inducing apoptosis and regulating cell cycle.

Furthermore, we investigated whether miR‐193a/b‐3p could reverse TGF‐β1 and activin A‐induced activation of LX‐2 cells. Our results showed that overexpression of miR‐193a/b‐3p significantly repressed TGF‐β1 and activin A‐induced increased COL1A1 and α‐SMA expressions. TGF‐β2 has been suggested to promote the expressions of ECM proteins.^35^ We also found that miR‐193a/b‐3p overexpression could inhibit TGF‐β1 and activin A‐induced expression of TGF‐β2 via directly targeting its 3′‐UTR. Smad2/3 has close relation with collagen gene expression. Su et  al indicated that inhibition of Smad2/3 phosphorylation could protect liver from fibrosis.^36^ The activation of HSCs could be restrained via regulating Smad2/3 pathway. Our results were consistent with previous studies and showed that miR‐193a/b‐3p overexpression restrained TGF‐β1 and activin A‐induced phosphorylation of Smad2/3. All the above results demonstrated that the activation of LX‐2 cells could be reversed by overexpression of miR‐193a/b‐3p.

Taken together, the up‐regulation of miR‐193a‐3p or miR‐193b‐3p alleviated ConA‐induced liver fibrosis through inducing apoptosis and cell cycle arrest of HSCs, and inhibiting the activation of HSCs. Although the detailed mechanisms need to be further elucidated, this study may shed light on how miR‐193a/b‐3p relieves ConA‐induced liver fibrosis.

## CONFLICTS OF INTEREST

The authors confirm that there are no conflicts of interest.
