# Supplementary figures and images for: miR‐193a/b‐3p relieves hepatic fibrosis and restrains proliferation and activation of hepatic stellate cells
Source: J Cell Mol Med. 2019 Apr 3;23(6):3824–32. doi: 10.1111/jcmm.14210 (PMC6533489; doi:10.1111/jcmm.14210)

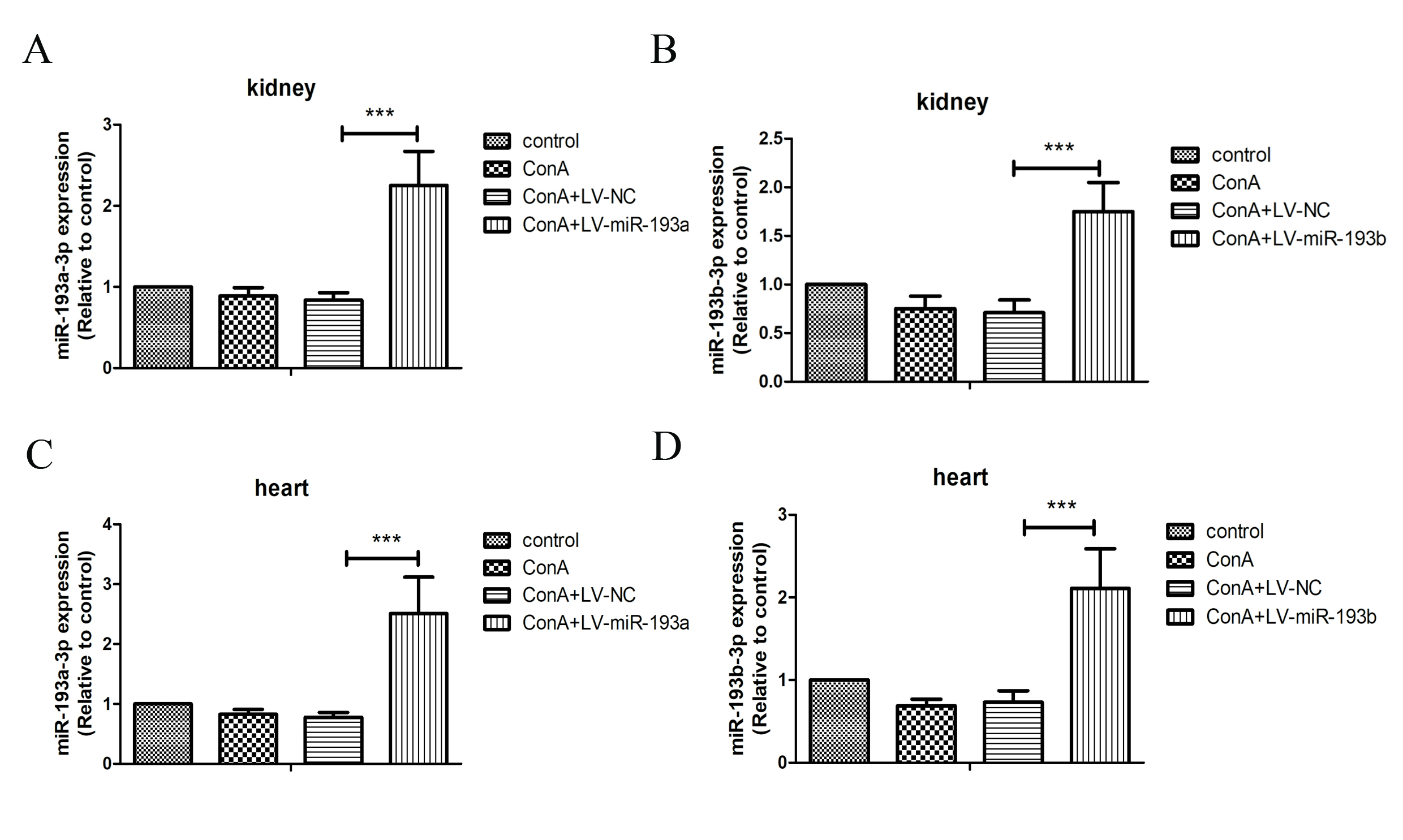

Supplement: Supplementary file 1 [file JCMM-23-3824-s001.tif]
