# Supplementary material for: miR‐193a/b‐3p relieves hepatic fibrosis and restrains proliferation and activation of hepatic stellate cells
Source: J Cell Mol Med. 2019 Apr 3;23(6):3824–32. doi: 10.1111/jcmm.14210 (PMC6533489; doi:10.1111/jcmm.14210)
Supplement: Supplementary file 2 [file JCMM-23-3824-s002.doc]

**Supplementary Figure 1** Expression of miR-193a/b-3p in kidney and heart tissues of mice. The expressions of miR-193a-3p (A&C) and miR-193b-3p (B&D) in kidney and heart tissues were detected by real-time PCR in different treatment groups. All data were expressed as mean ± SD (n=6). ***P<0.001 *versus* the indicated group.
